# The behavioral intention to adopt mobile health services: The moderating impact of mobile self-efficacy

**DOI:** 10.3389/fpubh.2022.1020474

**Published:** 2022-09-27

**Authors:** Isaac Kofi Mensah, Guohua Zeng, Deborah Simon Mwakapesa

**Affiliations:** ^1^School of Business Administration, Fujian Jiangxia University, Fuzhou, China; ^2^School of Economics and Management, Jiangxi University of Science and Technology, Ganzhou, China; ^3^School of Civil and Surveying Engineering, Jiangxi University of Science and Technology, Ganzhou, China

**Keywords:** e-health, e-health services, mobile health, mobile health services, mobile self-efficacy, UTAUT

## Abstract

This study explored the moderating impact of mobile self-efficacy on the adoption of mobile health services. The UTAUT was used as the theoretical foundation for this study. The results have indicated that mobile self-efficacy was significant in moderating the impact of both performance expectancy (β = −0.005, *p* < 0.05) and effort expectancy (β = −010, *p* < 0.05) on the adoption of mobile health services. In addition, it was revealed to our surprise that both performance (β = 0.521, *t* = 9.311, *p* > 0.05) and effort expectancy (β = 0.406, *t* = 7.577, *p* > 0.05) do not determine the behavioral intention to use mobile health services. Effort expectancy and behavioral intention to use were also, respectively, not significant in influencing performance expectancy (β = 0.702, *t* = 12.601, *p* > 0.05) and intention to recommend the adoption of mobile health services (β = 0.866, *t* = 13.814, *p* > 0.05). Mobile self-efficacy, however, was found to significantly predict the citizen's intention to recommend the adoption of mobile health services (β = 0.139, *t* = 2.548, *p* < 0.05). The implications of these findings on mobile health are discussed.

## Introduction

The strategic application of Information and communication technologies such as the internet to deliver public health services is known as electronic Heath (e-Health). E-health is considered as the use of ICT in the health sector for clinical, educational, research, and administrative purposes (1–4). E-health systems have the potential to facilitate the dissemination of health information and improve the access to information thereby creating greater awareness within the health care ecosystem (5–7). Hi-tech development in the IT sector has provided ample opportunities for the advancement in health and health care delivery systems thus making e-Health systems one of the major pillars in the health sector (5, 8). With the advancement in the development of the mobile communication technology industry, health delivery through an e-health system is shifting to mobile health systems (m-Health). Mobile health is gaining much attention due to the abundant availability of mobile phones and their related technologies such as 3G, 4G, and the recent 5G network launched by Huawei mobile company. These technologies particularly, the recent 5G-network presents opportunities to provide efficient mobile health services and solutions. M-heath is considered a spectacle attempt toward achieving healthcare innovation in the health sector (9–11).

Mobile health (m-Health) is defined as the application of mobile communications and network technologies to improve the delivery of healthcare systems (12–15). M-health systems ensure the delivery of healthcare without physical limitation and people can access health information and service anywhere and anytime regardless of their location (15–18). An efficient mobile health system has a positive effect on the nature of healthcare alerting and monitoring system, administrative data collection, maintaining health records, healthcare delivery activities, medical information awareness, and detection and prevention mechanisms (19–21). The development and deployment of the mobile health system can ensure some benefits such as reducing costs and providing conveniences for uses, reducing health services costs, reducing the isolation of users, and providing time for healthcare information and dissemination (22–24).

The development and deployment of mobile health services from the supply side are very simportant but equally important is the demand side perspective which explores the factors determining the adoption of mobile health services. Understanding the demand side of mobile health services is crucial for the e-health system to be considered successful since these factors provide direction and recommendations for public health policymakers to develop and implement an efficient m-healthcare service system that will be highly patronized. For instance, a study that examined citizens' behavior toward m-health services adoption in three different countries; the USA, Canada, and Bangladesh indicated differences and similarities in the significant factors determining the adoption of m-health services in these countries (25). Specifically, effort expectancy, facilitation conditions, price value, performance expectancy, social influence, and waiting time were all significant factors predicting the adoption of mobile health services in the USA, Canada, and Bangladesh (25). In the same study, while the social concept was a significant determinant in the case of both the USA and Canada, it however not significant in the context of the Bangladeshi citizens (25). Again, while Hedonic motivation was found to predict the adoption of mobile health services in Bangladesh it however not significant in determining the use of mobile health services among USA and Canadian citizens (25). Another study showed that trust, perceived usefulness, and perceived ease of use was a positive determinant of the intention to use m-Health services in China (26). The same study reported that privacy and performance risk were negatively related to patients' trust and behavioral intention to adopt m-Health services (26). Another study in Jordan demonstrated that perceived usefulness, perceived ease of use, awareness, and innovation were factors predicting the use of m-health services (27). In a related study, it was further validated that antecedents drive continued usage intention *via* the mediation role of e-satisfaction with m-health apps (28). The same research also reported that habit influences the continued usage intention and moderates the impact of e-satisfaction and continued intention of using m-health apps (28). These results are an indication of the culture-specific nature of the factors determining the adoption of mobile health services and thus the development and deployment of mobile health services by policymakers should be culture and country-specific. In others words, the policies guiding the development and deployment of mobile health services should be tailored to take into consideration the local and cultural context influencing the adoption of mobile health services.

One particular factor which has been examined in the context of mobile health services adoption is the issue of mobile self-efficacy (MSE). Mobile self-efficacy is an indispensable factor since it can determine the user's attitudes and decision to use mobile health services. Mobile self-efficacy (MSE) is the perception of individual users about their abilities to confidently navigate mobile devices (29–31) to access mobile health services. Mobile self-efficacy is not a necessary indicator of skill but rather an indicator of belief or confidence in one's skills or abilities to undertake or complete a particular course of action such as the use of mobile health services (32, 33). In the context of mobile health adoption studies have shown the positive effect of mobile self-efficacy on the adoption of mobile health services. For instance, it was demonstrated that self-efficacy is positively related to the perceived ease of use of mobile health services and also moderates the impact of perceived usefulness on the adoption of m-health services (34). Furthermore, self-efficacy was validated to influence people's attitudes toward the use of telemedicine (35).

The objective of this paper is to explore the moderating impact of mobile self-efficacy on citizens' adoption of mobile health services. This was done by integrating mobile self-efficacy into the Unified Theory of Acceptance and Use of Technology (UTAUT) developed by Venkatesh et al. (36). The UTAUT is considered the most used research theoretical underpinning for many information systems (IS) studies due to its integration with other eight major theories and for this current study, its utilization provides a robust foundation for the testing and validation of the proposed model in this paper and thus the achievement of the objective of this paper. This integration is expected to contribute to the mobile health literature by demonstrating the moderating impact of mobile self-efficacy on the relationship between both the performance and effort expectancy of mobile health services and the behavioral intention to adopt mobile health services. While studies have shown the direct impact of performance expectancy and effort expectancy on the behavioral intention to use mobile health services (37–42), no studies to the best of our knowledge have explored the moderating impact of mobile self-efficacy on these two relationships. This is the first attempted set of contributions to this study. The second contribution is the demonstration of the direct impact of mobile self-efficacy on the intention of citizens to recommend the adoption of mobile health services. This relationship between mobile self-efficacy and intention to recommend has also not been explored in the literature. The main research question to be investigated is: to what extent does mobile self-efficacy moderate the impact of performance expectancy and effort expectancy on the adoption of mobile health services?

The rest of the paper is ordered as follows: research theoretical framework and hypotheses, research model research, research methodology, results and data analysis, discussion, conclusion, and limitation of this study.

## Research framework and research hypotheses development

### The unified theory of acceptance and use of technology (UTAUT)

**Figure 1:**
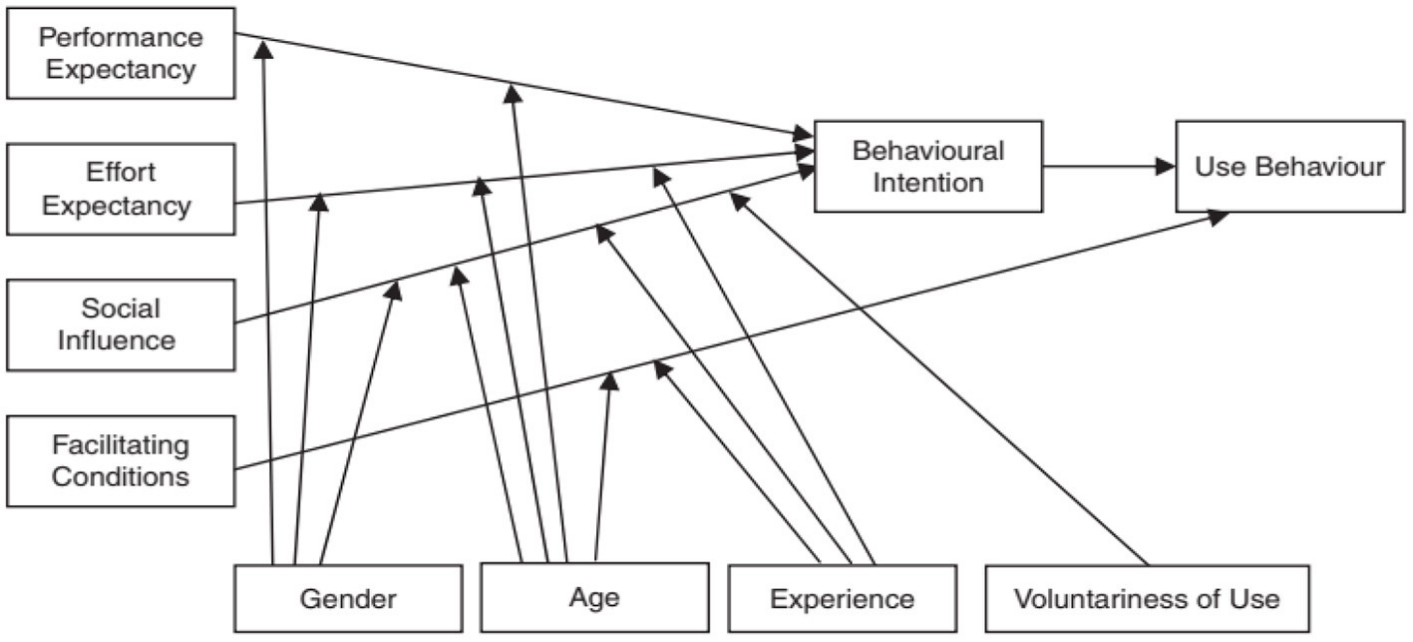
UTAUT (36).

The Unified Theory of Acceptance and Use of Technology (UTAUT) was developed through the integration of other technology adoption models (36). The UTAUT was developed by the combination of these theories: the Technology Acceptance Model (TAM), Theory of Reason Action (TRA), the Motivational Model, the Theory of Planned Behavior (TPB), combined TBP and TAM, the PC Utilization, Innovation Diffusion Theory (IDT), and Social Cognitive Theory (SCT). The integration of these models into the UTAUT is an attempt to provide a comprehensive model that can better explain the user behavioral adoption of new technologies. The UTAUT proposed four main constructs: performance expectancy, effort expectancy, social influence, and facilitating conditions which are considered to have a direct impact on the behavioral intention to use and the actual behavior as well (36). These four constructs are also moderated by gender, age, experience, and voluntariness of use (36). The UTAUT model is shown in Figure 1.

The UTAUT model has been applied by researchers and scholars over the years to identify factors influencing the acceptance of technology or innovation adoption across many fields of disciplines such as information systems, marketing, social psychology, and management (43, 44). Specifically, UTAUT has been utilized and validated in these fields like e-commerce (45–47), e-government (48–50), electronic health/mobile health (39, 51, 52), mobile banking (53–55), mobile payment/e-payment (56–58), and tourism (59, 60). These extensive applications and validations of the UTAUT provided confidence for its adoption in the current study.

### Performance expectancy (PE)

Performance expectancy is defined as the degree to which the individual user considers or perceives a technological system to be useful and beneficial in the performance of his or her job (36). Performance expectancy was determined to influence the behavioral intention to use (36). The application of technology in health care delivery would be adopted by citizens only if such technology adds to improving their quest to have access to quality and uninterrupted health services. Previous studies have demonstrated that performance expectancy has a direct impact on the adoption of mobile health services (37, 39, 61–64). Consequently, H1 was proposed.

H1: Performance expectancy of mobile health services is positively related to the intention to adopt mobile health services.

### Effort expectancy

The degree to which a technology is considered by individual citizens' to be easy to use and adopt is termed effort expectancy (36). Venkatesh et al. (36) have shown that effort expectancy had a positive effect on both the behavioral intention to use and performance expectancy. Mobile health applications that are designed with user-friendly tendencies or features would attract users to use and hence will have a subsequent effect on the decision of users to use it based on the performance expectancy of such a system. Studies have found a significant relationship between effort expectancy and behavioral intention to use e-health services as well as performance expectancy (37, 39, 42, 61, 65, 66). Accordingly, H2 and H3 were proposed.

**H2:** Effort expectancy of mobile health services is positively related to the intention to use mobile health services.

**H3:** Effort expectancy of mobile health services is positively related to the performance expectancy of mobile health services.

### Mobile self-efficacy

The development and availability of mobile technology have provided opportunities for citizens to use smartphones and tablets as a digital enhancement to their everyday activities (67–69). The mobile phone phenomenon has become an integral part of our modern life and in fact, it's part of our humanity since it has permeated all sectors of society from the aged to the young, work and unrelated work activities (70–72). Self-efficacy is considered one of the major factors underpinning the adoption of mobile phones or devices/new technologies (73–75). Self-efficacy is defined as the individual user's belief/confidence that they have the skills and capacity to undertake a particular action or behavior (29, 76). Mobile self-efficacy is therefore considered as the belief or confidence in the ability or capacity of individual users of mobile devices to operate mobile phone devices. Mobile self-efficacy concerning mobile health services is thus the citizens' confidence in their potentials or capacities/skills to operate mobile health service applications to acquire a specific type of health care service. Self-efficacy was found to be significant in the behavioral intention to adopt electronic health services and mobile health services (34, 77–79). In this current study, however, we seek to examine the moderating effect of mobile self-efficacy on both the impact of performance expectancy and effort expectancy on the adoption of mobile health services and also its direct impact on the intention of citizens to recommend mobile health adoption. Consequently, H4, H5, and H6 were proposed.

**H4:** Mobile self-efficacy plays a moderating role in the relationship between performance expectancy and the intention to use mobile health services.

**H5:** Mobile self-efficacy plays a moderating role in the relationship between effort expectancy and the intention to use mobile health services.

**H6:** Mobile self-efficacy is positively related to the intention to recommend the adoption of mobile health services.

### Behavioral intention to use

The UTAUT proposed the direct impact of the behavioral intention to use on the actual use of behavioral (36). Other recent studies also have validated the direct positive effect of intention to use on user behavior (37, 80). In this new study, we are proposing the direct impact of intention to use m-health services on the intention to recommend the adoption of m-health services. Previous studies have found a positive relationship between the intention to use and the intention to recommend (81, 82). Accordingly, H7 was proposed.

**H7:** Behavioral intention to use mobile health services is positively related to the intention to recommend the adoption of mobile health services.

## Research model

**Figure 2:**
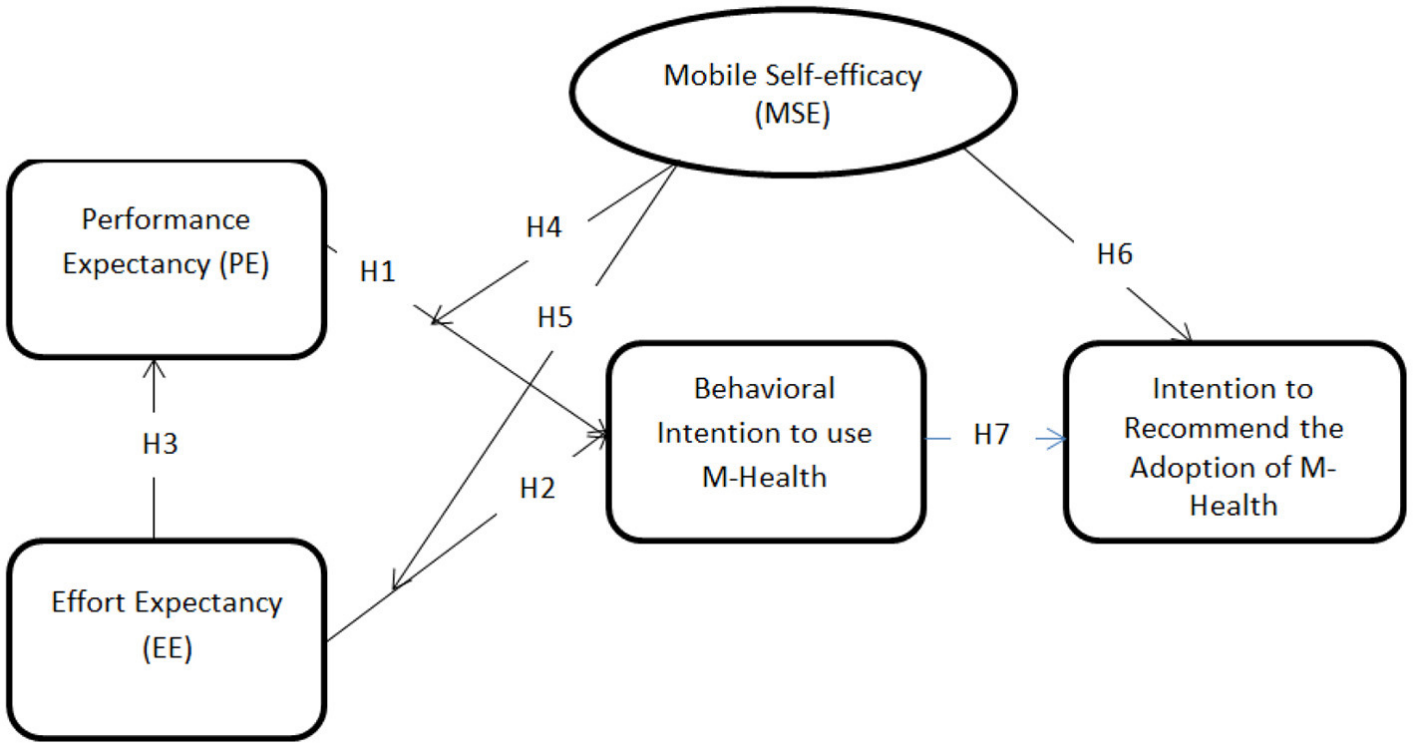
Proposed research model.

The research model based on the research hypotheses developed in the previous section is shown in Figure 2. Performance expectancy and effort expectancy are both projected to influence the intention to use mobile health services. Mobile self-efficacy is also presumed to moderate the impact of both performance and effort expectancy on the intention to use mobile health services. Also, the intention to use mobile health services is expected to influence the intention of citizens to recommend the adoption of mobile health services.

## Research methodology

A research questionnaire was used to generate data for this current study. The questionnaire items were selected based on a detailed literature review. Performance expectancy, effort expectancy, and behavioral intention to use were adopted from Venkatesh et al. (36) and Hoque and Sorwar (37), mobile self-efficacy from Eastin and Larose (83) and Lee and Lee (84), and intention to recommend from Oliveira et al. (81). The variable items used are shown in Table 1. The questionnaire instrument was developed in English and then translated into the Chinese language. This was done because first, the main language of the target population of this study is Chinese, and secondly to ensure that the potential respondents can resonate with the questionnaires to reduce any ambiguity there may have been. A five-point Likert scale of measurement ranging from 1= Strongly Disagree (SD) to 5 = Strongly Agree (SA) was used to measure the items of the questionnaire. The online platform was used as a method to administer the designed questionnaires to the targeted population of native citizens of Ganzhou city (specifically within the Zhanggong District), one of the cities in Jiangxi Province in China. After it was administered online for about three (3) and a half-month, a total of 306 responses were collected. The paper decided to go with the 306 samples collected since it meets the minimum sample required for the study. Using the sample size calculator, with a confidence level of 95%, a margin of error of 5%, a population proportion of 25%, and an estimated population size of 546,000, the minimum sample required is 288 (85). This is thus the justification for the use of the 306 valid responses acquired during the questionnaire administration process since it is more than the minimum required sample of 288, which is deemed to be an adequate sample size to conduct a statistical analysis. This sample size of 306 utilized is considered to be representative of the general population and thus reflects the characteristics of the population. The description of the sampled characteristics is shown in Table 2. SPSS and AMOS were used to capture and analyze the data generated through the use of structural equation modeling (SEM) techniques.

**Table 1:** Variable items utilized.

**Variables**	**Items**
**Performance Expectancy**	
PE1	I think m-health service is very useful
PE2	I think using m-health services will enable me to enjoy health care quickly
PE3	Using m-health services will increase my productivity
PE4	I can enjoy quality health care while using m-health services
PE5	Overall, I think m-health is good for me
**Effort Expectancy**	
EE1	I think learning to use m-health services is easy for me
EE2	I think my interaction with m-health services would be clear and understandable
EE3	I think m-health service is easy to use
EE4	I think it is easy for me to become skillful at using m-health services.
EE5	Overall, I think m-health services would not be hard to use
**Mobil Self-Efficacy**	
MSE1	I feel confident about understanding terms/words relating to m-health service.
MSE2	I feel confident about describing how to use m-health service
MSE3	I feel confident about troubleshooting m-health service problems
MSE4	I feel confident about using the m-health service to gather health data
MSE5	I feel confident about turning to an m-health service when help is needed
**Intention to Use**	
IU1	I will use m-health services in the future
IU2	I plan to use m-health services
IU3	I will try to use m-health services in the future
IU4	I intend to use m-health services frequently
IU5	overall, I think will have the continuous intention to use m-health services
**Intention to recommend**	
ITRCO1	I intend to recommend the use of m-health Services
ITRCO2	I will recommend to my close friends to use m-health services
ITRCO3	I will continue to recommend the use of m-health services
ITRCO4	Based on good experience with m-health services, I will recommend it
ITRCO5	If am satisfied with m-health services I will recommend it

**Table 2:** Respondent demographic profile.

**Item**	**Description**	**Frequency**	**Percentage %**
Gender	Male	124	40.52
	Female	182	59.48
age	18–25	259	84.64
	26–30	22	7.19
	31–35	5	1.64
	36+	20	6.53
Educational level	Under Graduate	250	81.70
	Masters	56	18.30
Salary per month	Under 2,000	236	77.12
	Over 2,000	70	22.88
MH usage experience	Yes	62	20.26
	No	244	79.74
Mobile phone usage experience	1–4 year	142	46.40
	5 year+	164	53.60

## Results and data analysis

### Respondents' demographic profile

The basic information about the respondents that were gathered during the question administration is shown in Table 2. Many of the respondents were female (59.48%) while the largest age distributions of the respondents were between the ages of 18–25 years (84.64%). The majorities of the respondents were undergraduate students (81.70%) and had earned salaries under 2000 RMB (about 300 Dollars). While only 20.26 % of the respondents indicated (YES) that they had used mobile health service, 79.74% indicated (NO) that they had not used it before. In terms of the experience of using smartphones, the majority indicated that they had 5 years and above experience (53.60%).

### Descriptive statistics

The descriptive statistics of the data are shown in Table 3. The descriptive statistics are displayed to indicate the mean and standard deviations for the constructs used in this study. The means of the constructs appear to be closer to 5 which meant that respondents responded favorably to the instrument. Also, there was a significant positive correlation between all the constructs examined. The outcome of the descriptive statistics met our expectations and hence, therefore, provided the basis to continue with the data analysis.

**Table 3:** Descriptive statistics.

**Variable**	**Mean**	**Standard deviation**	**PE**	**EE**	**MSE**	**BIU**	**IR**
PE	3.68	0.839	1				
EE	3.58	0.887	0.660**	1			
MSE	3.565	0.8608	0.729**	0.725**	1		
BI	3.725	0.8364	0.749**	0.729**	0.754**	1	
RI	3.695	0.8369	0.740**	0.689**	0.710**	0.847**	1

Performance Expectancy (PE), Effort Expectancy (EE), Mobile Self-Efficacy (MSE), Behavioral Intention to Use (BIU), and intention to recommend (IR). ** indicates a significant correlation at the 0.01 (two-tailed) level of significance.

### Measurement model

The results of the measurement model are shown in Table 4. The average variance extracted (AVE), composite reliability (CR), factor loadings, and Cronbach alpha were used as the quality criterion to determine the quality of the measurement model. The acceptable values for Cronbach's alpha and factor loadings for each item are 0.70 and above (86, 87). In addition, the recommended and acceptable value for composite reliability is 0.70 and the average variance extracted is 0.50 (87, 88). As indicated in Table 3, all the recommended and acceptable values, respectively, for composite reliability, average variance extracted, factor loadings, and Cronbach alpha have been achieved. This is an indication that the first quality criterion for the measurement model has been met.

**Table 4:** Construct validity and reliability analysis.

**Constructs**	**Code**	**Factor Loadings**	***T*-value**	**Standard factor load**	**AVE**	**CR**	**Cronbach's alpha**
Performance expectancy	PE1	1.000		0.844	0.7515	0.9379	0.928
	PE2	0.978	19.754	0.870			
	PE3	1.057	21.715	0.918			
	PE4	0.963	18.329	0.834			
	PE5	0.982	19.546	0.866			
Effort expectancy	EE1	1.000		0.875	0.7934	0.9505	0.900
	EE2	0.969	22.982	0.897			
	EE3	0.983	22.426	0.892			
	EE4	0.988	23.592	0.914			
	EE5	0.963	21.422	0.875			
Mobile self-efficacy	MSE1	1.000		0.906	0.7682	0.943	0.913
	MSE2	0.963	24.509	0.894			
	MSE3	0.961	22.807	0.872			
	MSE4	0.946	21.414	0.855			
	MSE5	0.951	21.342	0.854			
Behavioral intention to use	BI1	1.000		0.894	0.7731	0.944	0.904
	BIU2	0.993	23.379	0.888			
	BIU3	1.037	25.310	0.910			
	BIU4	0.930	19.594	0.825			
	BIU5	0.971	22.344	0.877			
Intention to recommend	IR1	1.000		0.902	0.7316	0.9314	0.900
	IR2	0.975	25.273	0.881			
	IR3	0.987	26.279	0.892			
	IR4	0.929	22.496	0.841			
	IR5	0.880	18.195	0.752			

The next was to determine the extent of discriminant validity that exists in our constructs. The discriminant validity was determined by using the Fornell-Larcker criterion. This principle indicates that discriminant validity is said to occur or exist if the square of the AVE is greater than the paired inter-correlations between the latent constructs (89). The results of the discriminant validity conducted are shown in Table 4. As illustrated in Table 5, the crosswise variables (square root of the AVE) are greater than its equivalent off-crosswise variables (paired inter-correlations) which meant that the Fornell-Larcker principle has been met. This is a further indication of the discriminant validity of the constructs used in this study.

**Table 5:** Discriminant validity.

**Constructs**	**PE**	**EE**	**MSE**	**BIU**	**IR**
PE	**0.8669**				
EE	0.660**	**0.8907**			
MSE	0.729**	0.725**	**0.8765**		
BIU	0.749**	0.729**	0.754**	**0.8793**	
IR	0.740**	0.689**	0.710**	0.847**	**0.8553**

Fornel-Lacker Criterion: Matrix of correlation constructs and the square root of AVE (in bold). **indicates a significant correlation at the 0.01 (two-tailed).

### Structural model

#### Direct relationships

The results of the research hypotheses (direct effects) tested are shown in Table 6. The results indicated that performance expectancy was not a significant predictor of the behavioral intention to use mobile health services (β = 0.521, *t* = 9.311, *p* > 0.05). H1 was therefore not supported. It also found that effort expectancy was not significant determinate of both performance expectancy (β = 0.702, *t* = 12.601, *p* > 0.05) and behavioral intention to use mobile health services (β = 0.406, *t* = 7.577, *p* > 0.05). Accordingly, H2 and H3 were also not supported. It was however discovered that mobile self-efficacy was a significant predictor of the intention to recommend the adoption of mobile health services (β = 0.139, *t* = 2.548, *p* < 0.05). H4 was hence supported. Also, it was shown that the behavioral intention to use does not determine significantly the intention to recommend the adoption of mobile health services (β = 0.866, *t* = 13.814, *p* > 0.05). H5 was thus not supported.

**Table 6:** Research hypotheses tested-direct effect.

**Hypotheses**	**Path**	**β**	***T*-value**	**Supported**
H1	PE → BIU	0.521	9.311	NO
H2	EE → PE	0.702	12.601	NO
H3	EE → BIU	0.406	7.577	NO
H4	MSE → IR	0.139	2.548	YES
H5	BI → IR	0.866	13.814	NO

#### Moderating effects

**Figure 3:**
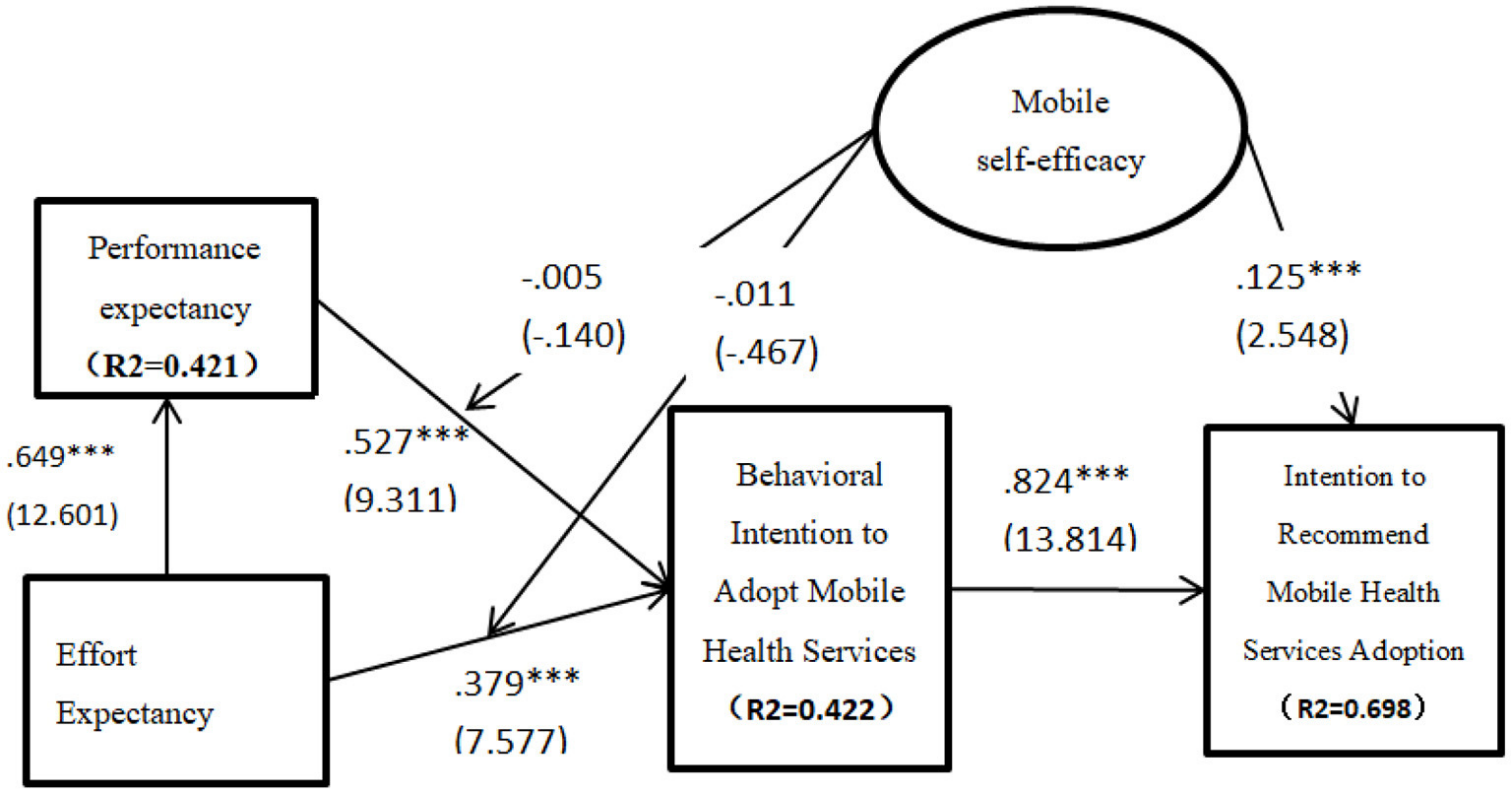
Validated structural model.

The analysis of the moderating effects is shown in Table 7 while the summary of these moderating relationships is shown in Table 8. The summary of the moderating relationships shown in Table 8 indicates that the moderating relationships tested were statistically supported. Specifically, it was found that there was a significant moderating effect of mobile self-efficacy on the relationship between performance expectancy and behavioral intention to use (β = −0.005, *p* < 0.05). Hence H6 was supported. Again it was discovered that mobile self-efficacy moderates significantly the impact of effort expectancy on the behavioral intention to use mobile health services (β = −010, *p* < 0.05). H7 was accordingly supported. The validated structural model depicting both direct and indirect relationships is shown in Figure 3.

**Table 7:** Moderation effects.

**变量**	**Model1**	**Model2**	**Model3**	**Model4**	**Model5**
MAN	0.751*** (0.039)	0.441*** (0.050)	0.441*** (0.050)	0.289*** (0.056)	0.284*** (0.057)
U25AGE	0.045 (0.038)	0.057* (0.034)	0.057* (0.034)	0.041 (0.033)	0.043 (0.033)
UCOLL	0.004 (0.058)	0.020 (0.052)	0.020 (0.052)	0.041 (0.050)	0.042 (0.050)
STUD	−0.055 (0.042)	−0.086** (0.038)	−0.087** (0.038)	−0.092** (0.036)	−0.092** (0.036)
U2000	0.006 (0.081)	−0.047 (0.073)	−0.048 (0.073)	−0.066 (0.070)	−0.069 (0.070)
UMHS	−0.073 (0.071)	−0.047 (0.064)	−0.046 (0.064)	−0.030 (0.061)	−0.032 (0.061)
NU	−0.064 (0.039)	−0.127*** (0.035)	−0.127*** (0.036)	−0.116*** (0.034)	−0.115*** (0.034)
B14YEAR	0.017 (0.039)	−0.001 (0.035)	−0.001 (0.035)	−0.001 (0.033)	0.002 (0.034)
MSE		0.434*** (0.051)	0.433*** (0.051)	0.365*** (0.050)	0.367*** (0.051)
PE		−0.016 (0.036)	−0.017 (0.036)		
MPE			−0.005 (0.035)		
EE				0.271*** (0.051)	0.267*** (0.052)
MEE					−0.010 (0.023)
_CONS	9.141E-7 (0.037)	2.112E-7 (0.034)	−0.001 (0.034)	7.893E-9 (0.032)	0.007 (0.035)
N	306	306	306	306	306
R2	0.583	0.666	0.666	0.695	0.695
Adjusted R2	0.572	0.655	0.654	0.685	0.684
F	51.925	53.328	58.854	67.282	61.017
VIF	≤ 4.663	≤ 4.698	≤ 4.701	≤ 4.709	≤ 4.735

*0.001, **0.01, ***0.05.

**Table 8:** Summary of the moderation effect.

**Hypotheses**	**Path**	**β**	***T*-value**	**Supported**
H6	PE*MSE → BI	−0.005	−0.140	YES
H7	EE*MSE → BI	−0.010	−0.467	YES

The validated structural model of this study is shown in Figure 3. The model explains about 42.2% and 69.8%, respectively, of the behavioral intention to adopt mobile health services and the intention of citizens to recommend the adoption of mobile health services.

## Discussion

This study adopted the unified theory of acceptance and use of technology (UTAUT) to explore the adoption of mobile health services among citizens in the city of Ganzhou, in Jiangxi Province of China. The UTAUT was integrated with mobile self-efficacy as moderating construct; moderating the impact of both the main constructs of the UTAUT i.e., performance expectancy and effort expectancy on the behavioral intention to use mobile health services. In addition to mobile self-efficacy, the intention to recommend was also introduced into the UTAUT model. To our surprise, the result of the study has shown that both the main constructs of the UTAUT, performance expectancy and effort expectancy were both not significant predictors of the intention to use mobile health services. The non-significant impact of performance and effort expectancy on the behavioral intention to use does not support previous studies which have indicated the positive effect of these two factors (PE and EE) on the adoption of mobile health services (38, 39, 41, 66, 90, 91). Other studies, however, have corroborated our findings of the non-significant impact of performance expectancy and effort expectancy on the intention to adopt (51, 92). In addition, other previous findings have also illustrated that performance (93–95) and effort expectancy (96–99) does not determine the intention to use. The reasons accounting for the non-significant performance expectancy and effort expectancy on the behavioral intention to adopt mobile health services could be due to first, the advanced technology savvy of citizens along with its long usage and hence they have become skilled at the use of technology related applications. This thus makes them able to have easy access to mobile technology services and hence the issue of ease of use accompanying the use of mobile health services becomes less of a challenge. Our view is supported by Yuan et al. (98) who indicated that the advancement in the development of smartphone interfaces concerning its usability, reduces the degree of effort citizens may encounter in the use of mobile health services. Secondly, the lack of awareness among the population sampled concerning mobile health services may explain the non-significant impact of performance expectancy on the intention to use. Proper awareness creation about mobile health services is important since it serves as an informational tool to educate citizens about the benefits that will accrue in the use of mobile health services. Knowledge about these benefits and the kinds of health services that they stand to enjoy from mobile health will have a positive effect on their understanding of the performance expectancy of mobile health services. This view is also shared by Hossain et al. (51) who elaborated that the non-acquaintance of users with e-health applications and their benefits may account for the non-significant performance expectancy on the intention to adopt.

In addition, effort expectancy does not determine the performance expectancy of mobile health services. The non-significant impact of effort expectancy on performance expectancy does not correspond to previous studies that showed a significant positive relationship between effort and performance expectancy (100, 101). Another interesting result was that the behavioral intention to use mobile health services does not influence the intention of citizens to recommend the adoption of mobile health services. Our finding is a departure from other similar studies which have demonstrated that the behavioral intention to use does determine the intention to recommend a technology (81, 82). This result may be an indication that just the citizen's intention to use MHS will not automatically lead to its recommendation to others to also use or adopt because they may not have used or had any experiences with the MHS services and thus will not be interested to recommend something they have had no personal experiences with. It also means that recommendations should not just be based on the perceptions (intentions to use) but on knowledge and experience arising from its usage which can either be satisfactory or unsatisfactory. So upon these actual usage experiences (satisfactory or unsatisfactory) users can then be in a better position to either decide to recommend the use of mobile health services to others or not. To buttress our point, as indicated in Table 2, the majority of the respondents (79.74%) indicated that they have had no experience using mobile health services. This may have resulted in the ultimate decision not to recommend the adoption of mobile health services which this study has empirically supported.

Furthermore, the results have demonstrated that mobile self-efficacy is positively related to the behavioral intention to recommend the adoption of mobile health services. In exploring the indirect effect of the study (moderating effect of mobile self-efficacy), it was shown that mobile self-efficacy was significant in moderating the impact of both performance expectancy and effort expectancy on the adoption of mobile health services. What these results mean is that, though performance expectancy and effort expectancy were not significant in determining the intention to use mobile health services, the inclusion of mobile self-efficacy, however, contributes positively to strengthening the predictive power of both performance expectancy and effort expectancy on the behavioral intention to use mobile health services. It further implies that citizen's confidence in their ability to use and operate mobile phones/smartphones has the potential to first of all influence their intention to recommend the adoption of mobile health services to others and secondly, improve their perceptions of usefulness and easiness associated with mobile health services which would indirectly affect their intention to use mobile health services. These findings on moderating effect of mobile self-efficacy are empirical attestations of the important role mobile self-efficacy can play in the adoption of mobile health services as citizens seek to access and enjoy quality health services.

The significant moderating effect of mobile self-efficacy on the impact of performance and effort expectancy on the behavioral intention to adopt m-health services could not be compared with any previous study. This thus demonstrates the contribution and novelty of the study to the m-health literature as no study has validated these relationships. For instance self–efficacy was found to have a positive impact on the effect expectancy (80) but did not experiment on the moderating impact of mobile self-efficacy on the relationship between effort expectancy and intention to use. Also, self-efficacy showed a direct positive impact on performance expectancy and effort expectancy of mobile health services (52), however again, it did not explore the extent to which mobile self-efficacy can indirectly (moderate) the influence of these core variables (performance and effort expectancy) of the UTAUT on the behavioral intention to use mobile health services.

### Theoretical implications

The first theoretical implication is that mobile self-efficacy moderates significantly the impact of both performance expectancy and effort expectancy on the behavioral intention to adopt mobile health services. Secondly, mobile self-efficacy is a positive predictor of the intention to recommend the adoption of mobile health services. Additionally, the key constructs of UTAUT i.e., performance expectancy and effort expectancy jointly accounted for 42.2% of the reasons driving the adoption of m-health services. Effort expectancy accounted for 42.1% of the factors influencing the performance expectancy of m-health services. Finally behavioral intention to use accounted for 69.8% of the elements making up the citizens' intention to recommend the adoption of m-health services. These two theoretical implications are an important extension of the UTAUT model and therefore contribute to the mobile health adoption literature.

### Practical implications

Mobile technology has been the most solicited method recently in the health technology industry to deliver quality health services and care to people 24/7 and particularly to reach places where there is no health care facility or services due to the remoteness of these regions. While the availability of mobile phones is important for mobile health to be diffused, the mobile self-efficacy of the people owning these smartphones is equally important. Since a person with low levels of self-efficacy may be impeded to access health services delivered through mobile/smartphones. As this study has indicated, the extent of citizens' mobile self-efficacy can influence the adoption of mobile health services. Service providers, government, and public health practitioners must, therefore, pay attention to not just the availability of affordable smartphones but also the empowerment of citizens with the right skills and capabilities (mobile self-efficacy) to be able to access mobile health services through their phones. These skills and abilities can improve the mobile self-efficacy of citizens which would, in turn, encourage them to recommend the adoption of mobile health services to their colleagues, family, and friends. The recommendation of mobile health services to friends and colleagues is an important step toward the successful diffusion of m-health services.

In addition, increasing the confidence of citizens through mobile self-efficacy can also have an indirect influence on their perception of the performance and effort expectancy toward the adoption of mobile health services. This is because this study has demonstrated that mobile self-efficacy can strengthen the impact of both performance expectancy and effort expectancy on the adoption of mobile health services. Once citizens achieve a good level of mobile self-efficacy then the issue of easiness and performance of mobile health services will not be a problem for them to use health services delivered to them through their smartphones.

## Conclusions

This research paper investigated the moderating effect of mobile self-efficacy on the adoption of mobile health services based on UTAUT as its theoretical foundation. The data analysis has supported the significant moderating effect of mobile self-efficacy on the relationship between performance expectancy and effort expectancy on the behavioral intention to use mobile health services. The empirical support for the moderating effect of mobile self-efficacy tested is the major objective and findings of this study. Also, mobile self-efficacy was a significant predictor of the intention to recommend the adoption of mobile health services. These two findings are the major findings and contributions of this study to the mobile health adoption literature. Additionally, the findings of this current study have provided policy implications and direction for governments, health service providers, and public health practitioners to pay more attention to the mobile self-efficacy of citizens since the lack or absence of higher levels of mobile self-efficacy can affect negatively the adoption of mobile health services and its diffusion. Particularly, in developing countries and regions where smartphone usage is rare, issues of mobile self-efficacy would be a critical matter to deal with.

## Limitations and future research

The sample size is the first limitation of this study and hence the results and findings of this study should not be over-generalized. Secondly, the models and the constructs examined in this study could be applied in the context of other developing countries and the results may not reflect or support the findings of this study. Not all the factors driving the adoption of mobile health services were examined in this study since no single study can do so. Consequently, future studies will endeavor to examine the direct effect of mobile self-efficacy on the main constructs of the UTAUT model and also explore the cost of the mobile bundle on the adoption of mobile health services.

## Data availability statement

The original contributions presented in the study are included in the article/supplementary material, further inquiries can be directed to the corresponding author/s.

## Ethics statement

Ethical review and approval was not required for the study on human participants in accordance with the local legislation and institutional requirements. Written informed consent for participation was not required for this study in accordance with the national legislation and the institutional requirements.

## Author contributions

IM: conceptualization, methodology, data collection, analysis, and writing—original draft preparation. GZ: Writing and literature review, revisions, and secure funding. DM: literature review, revisions, and editing. All authors contributed to the article and approved the submitted version.

## Conflict of interest

The authors declare that the research was conducted in the absence of any commercial or financial relationships that could be construed as a potential conflict of interest.

## Publisher's note

All claims expressed in this article are solely those of the authors and do not necessarily represent those of their affiliated organizations, or those of the publisher, the editors and the reviewers. Any product that may be evaluated in this article, or claim that may be made by its manufacturer, is not guaranteed or endorsed by the publisher.
